# Nanoclays: Promising Materials for Vaccinology

**DOI:** 10.3390/vaccines10091549

**Published:** 2022-09-17

**Authors:** Dania O. Govea-Alonso, Mariano J. García-Soto, Lourdes Betancourt-Mendiola, Erika Padilla-Ortega, Sergio Rosales-Mendoza, Omar González-Ortega

**Affiliations:** 1 Facultad de Ciencias Químicas, Universidad Autónoma de San Luis Potosí, Av. Manuel Nava 6, Zona Universitaria, San Luis Potosí 78210, Mexico; 2 Sección de Biotecnología, Centro de Investigación en Ciencias de la Salud y Biomedicina, Universidad Autónoma de San Luis Potosí, Av. Sierra Leona 550, Lomas 2ª. Sección, San Luis Potosí 78210, Mexico

**Keywords:** adjuvanticity, nanocarrier, bioconjugation, hectorite, layered double hydroxides, halloysite nanotubes

## Abstract

Clay materials and nanoclays have gained recent popularity in the vaccinology field, with biocompatibility, simple functionalization, low toxicity, and low-cost as their main attributes. As elements of nanovaccines, halloysite nanotubes (natural), layered double hydroxides and hectorite (synthetic) are the nanoclays that have advanced into the vaccinology field. Until now, only physisorption has been used to modify the surface of nanoclays with antigens, adjuvants, and/or ligands to create nanovaccines. Protocols to covalently attach these molecules have not been developed with nanoclays, only procedures to develop adsorbents based on nanoclays that could be extended to develop nanovaccine conjugates. In this review, we describe the approaches evaluated on different nanovaccine candidates reported in articles, the immunological results obtained with them and the most advanced approaches in the preclinical field, while describing the nanomaterial itself. In addition, complex systems that use nanoclays were included and described. The safety of nanoclays as carriers is an important key fact to determine their true potential as nanovaccine candidates in humans. Here, we present the evaluations reported in this field. Finally, we point out the perspectives in the development of vaccine prototypes using nanoclays as antigen carriers.

## 1. Introduction

Vaccines are valuable tools to fight against human and animal diseases, primarily infectious ones, but over the last decades they have also been intensively focused against noncommunicable diseases. However, the optimal exploitation of vaccines to promote global health requires the development of vaccines against neglected diseases and fast-track processes to achieve their approval in record time for vaccines against emerging pathogens. The time aspect is a special need for developing countries that often depend on developed countries to obtain such vaccines, dealing not only with non-priority availability of the vaccines but also with budget restrictions to afford them. Moreover, storage and distribution of most vaccines is cold-chain dependent, which increases the cost for massive vaccination campaigns. Therefore, the development of proprietary vaccine platforms, seeking thermal stability and meeting high safety and efficacy is a key goal for such countries [1].

The development of subunit vaccines requires not only the identification of a protective antigen, but also a formulation to induce robust immune response against such antigen. This is critical, as such vaccines inherently have a simplified composition (yielding poor immunogenic activity) with respect to those based on the whole pathogen [2]. Particulate antigen delivery systems are an alternative to enhance the antigen immunogenicity through several mechanisms, including enhanced recognition and uptake by antigen presenting cells, intrinsic immunostimulatory activities, and enhanced antigen stability and bioavailability as consequence, in part, of higher tissue penetration capacity [3]. The advent of nanotechnology opened new routes for the development of particulate antigen delivery systems. Both organic and inorganic particles have been evaluated to determine their potential to deliver antigens safely and efficiently in mammals and other target organisms, with a subsequent robust immune response relative to using the antigen alone or its conventional co-administration with adjuvants [4]. Until now, in terms of vaccines for human use, liposomes and virus-like particles are successful examples of nanovaccines based on organic carriers since these are used to formulate COVID-19 and HPV vaccines currently applied worldwide [5]. PLGA (poly(lactic-co-glycolic acid)) particles are another example of organic particles [6].

The application of inorganic nanoparticles in vaccinology is behind their organic counterparts. Although gold, calcium carbonate, and other inorganic materials have proven adjuvanticity in a variety of vaccine prototypes, few cases have progressed into clinical trials and thus far no vaccines based on these carriers have achieved approval for human use. However, it is of particular interest to note that alum, a conventional inorganic adjuvant formed by microparticles, has been recently explored in the nanometric scale, leading to promising findings, since the nanosized adjuvant favored the induction of a more balanced immune response (Th1/Th2) with respect to the conventional microparticles. This effect is expected to lead to vaccines with enhanced efficacy and safety. For instance, cancer and other diseases (such as infections caused by intracellular pathogens) require Th1 responses to be effectively resolved. Moreover, the induction of a Th2-biased response induced by the microparticles is associated with the induction of side effects (hypersensitivity) in atopic individuals [7].

In this context, nanoclays are attractive platforms to be validated as antigen carriers with immunostimulatory activity. Considering that the expansion on the assessment of new materials will undoubtedly offer new possibilities for the development of innovative vaccines, both synthetic and natural clays should be systematically evaluated as antigen carriers. Some research groups have focused on the characterization of synthetic clays as antigen carriers, while the use of natural clays remains scarce. The adsorption of proteins on clay materials has been studied with several uses in agriculture [8], bioremediation [9,10], and biomedicine. In this last, drug delivery [11], tissue engineering [12,13], and vaccination [14,15] have been investigated. The use of clays as nanocarriers has numerous advantages, including low cytotoxicity, biocompatibility, simple particle-size control, feasible functionalization, and low costs [16]. Inorganic components such as aluminum hydroxide derivatives are used as vaccine carriers. Nevertheless, these components have numerous disadvantages, such as poor thermal and freeze-drying stability, and susceptibility to agglomeration that can cause damages to the antigen structure, affecting its immunogenic potential [15]. The development of new carriers that can bring higher stability to the biohybrid and enable a simple administration is an actual challenge. In this context, clays minerals are an excellent option due to their intrinsic characteristics combined with their large protein adsorption capacity. In recent years, some uses of clay as a vaccine adjuvant have been reported [14,17].

This review aims to present an integral view on the role of nanoclays in vaccinology, covering their chemical description, methods for their synthesis and functionalization with specific moieties and/or antigens, and the state of the art of evaluating vaccine prototypes at the preclinical level, as well as identifying key perspectives for the field.

## 2. Clays Minerals and Nanoclays

**Figure 1:**
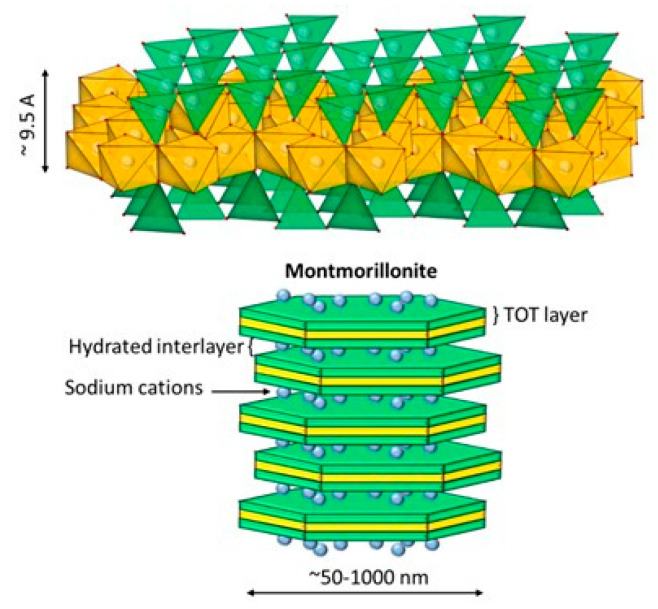
Molecular structure of montmorillonite containing exchangeable sodium [23].

Clay minerals are considered a valuable natural resource since they are minerals widely available in the Earth’s crust. Such clays have been used since ancient times as raw materials for ceramics. Nowadays, they are used in a wide variety of products that includes paints, cosmetics, and pharmaceuticals. Their large specific surface area (50–300 m^2^/g), chemical, mechanical, and thermal stability, along with their layered structure, high cation-exchange capacity (CEC) (20–300 meq/100 g), and the presence of Brönsted and Lewis acid sites on the surface, make clay minerals excellent adsorbent materials [18]. Clays comprise a group of minerals, mostly phyllosilicates, whose crystal structure is based on sheets arranged in layers. The individual layers are composed of two, three, or four sheets that can be tetrahedral (T) and octahedral (O). The tetrahedral sheets are composed of tetrahedral groups of silicon that are linked by sharing three of their four oxygens with other tetrahedra. The octahedral sheets are formed from octahedral [AlO_3_(OH)_3_]_6_ groups, which are assembled by sharing one of their six vertices with the vertex of another octahedron [19,20]. Clays are characterized by having reactive groups on their edges due to the presence of hydroxyl groups (silanols and aluminols). Due to the charge imbalance that exists in their layers, they have cation exchange capacity. Figure 1 shows the structure of montmorillonite, which is a TOT (2:1) clay with sodium as interchangeable cation. Clays have been investigated and employed in environmental, biomedical, and pharmaceutical fields [21]. These applications include adsorption of heavy metals from surface and groundwater, and the development of clay-based nanoparticles and biohybrid materials.

Clay minerals can be classified based on their structural arrangement into layered, fibrous, and tubular clays. The most representative clays of these groups are bentonite (montmorillonite), sepiolite, and halloysite nanotubes, respectively. Natural clays can be classified within 2D materials due to the interlayer space and crystal size. Nanoclays consist of approximately 1 nm thick alumina silicate layer surface that ensembles about 10 nm multilayer stacks. Compared to clays, nanoclays exhibit several advantageous properties that include higher workability, mechanical strength, and heat resistance [22].

### 2.1. Halloysite

**Figure 2:**
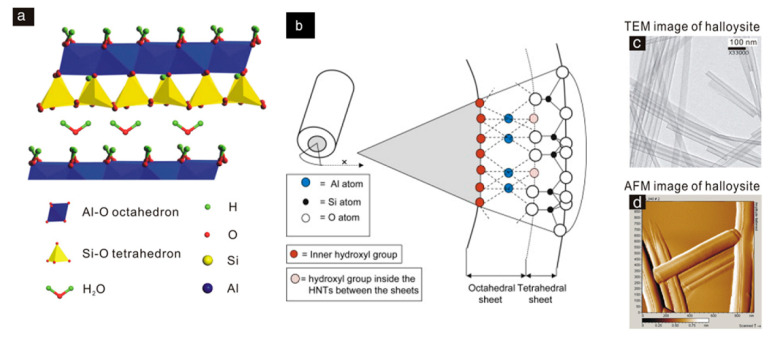
Halloysite nanotubes (HNT). (**a**) HNT crystal morphology and (**b**) atomic structure, (**c**) TEM (micrograph of transmission electron microscopy), and (**d**) AFM (atomic force microscopy) images [30].

Halloysite nanotubes (HNT), consisting of rolled aluminosilicate layers, are inert clays with a theoretical formula similar to kaolinite: Al_2_Si_2_O_5_(OH)_4_·*n*H_2_O [24]. The hydrated form of halloysite (when *n* = 2) is named “halloysite-(10 Å)”. In this structure, the multilayers are separated by a monolayer of water molecules with a basal d001 spacing of 10 Å. The dehydrated structure of halloysite (when *n* = 0) is named “halloysite-(7 Å)” [25]. The hollow cylindrical structure of the halloysite nanotube and the water between the nanotube interlayers is one of the most important characteristics that distinguish it from kaolinite [25]. The outer surface diameter, the inner cavity diameter, and length of HNT typically vary between 50–100 nm, 10–30 nm, and 100–2000 nm, respectively. The outer surface of HNT has different chemical and electrical properties from the inner surface [26]. Structurally, the outer surface consists of a silicon dioxide (SiO_2_) tetrahedral layer, whereas the inner cylinder core consists of an alumina layer (Al_2_O_3_). They contain inner and outer hydroxyl groups located between the layers. The outer surface has a lower density of hydroxyl groups (Si-OH) and is negatively charged, while the inner surface has a larger amount of hydroxyl groups (Al-OH) and is positively charged [27]. The outer surface of HTN is occupied by Si–O–Si groups, while Si–OH groups that are located on the edges of the layers (Figure 2). Therefore, the strong electronegativity of oxygen atoms makes the outer surface negatively charged in the 2.0–12.0 pH range [28,29].

### 2.2. Layered Double Hydroxides

**Figure 3:**
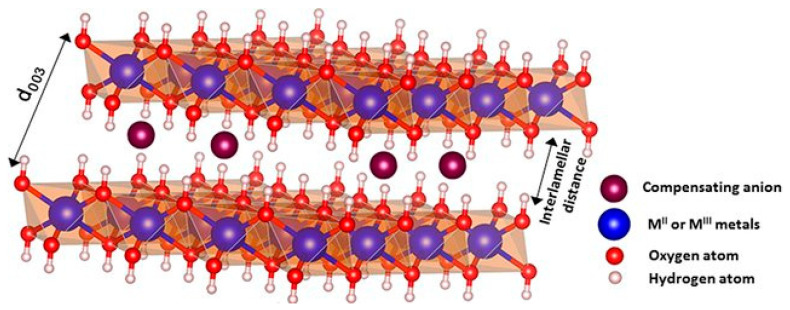
LDH atomic structure [39]. With permission.

Layered double hydroxides (LDH), also known as anionic clays or “hydrotalcite-like”, have interesting characteristics. When compared to natural clays, LDH have the advantage that their composition can be controlled, along with their dimensions. Figure 3 shows the typical structure of LDH. LDH are represented by the general formula [M1−xIIMxIII(OH)2]x+[Axnn−]×yH2O, where $M^{\mathrm{II}}$ indicates divalent metal cations, such as Mg^2+^, Ca^2+^, Zn^2+^, Cu^2+^, Co^2+^, or Ni^2+^, and $M^{\mathrm{III}}$ denotes trivalent metal cations, such as Fe^3+^, Al^3+^, Ga^3+^, or Cr^3+^. The electroneutrality of LDH is achieved by the presence of hydrated organic or inorganic $A^{n-}$ anions, such as ${\mathrm{CO}}_{3}^{2-}$, ${\mathrm{NO}}_{3}^{-}$, ${\mathrm{SO}}_{4}^{2-}$, OH^−^, Cl^−^, or Br^−^ in the interlamellar space. Over the last decades, many studies have dealt with the synthesis of LDH. Some methods are easy to follow and allow developing low-cost and eco-friendly LDH. In this regard, LDH can be obtained by coprecipitation [31,32], sol–gel methods [33], hydrothermal synthesis [34,35], urea hydrolysis [36], ion exchange, calcination-rehydration, self-assembly, hydrothermal/solvothermal, in situ chemical reduction, and mechanochemical procedures. Karim et al. [37] summarized all these methods. The synthesis of LDH by coprecipitation is the simplest, cheapest, and most used method for obtaining LDH with high purity and crystallinity. Moreover, the resulting products are composed of LDH particles aggregated with a particle size of 10–50 µm. On the other hand, compared to the coprecipitation method, the hydrothermal technique enables the synthesis of LDH with uniform morphology and highly crystalline structure [37]. In recent years, a two-step synthesis (coprecipitation–hydrothermal) has been optimized, resulting in LDH nanoparticles with size between 60 and 150 nm, with good yields and crystallinity [38].

### 2.3. Hectorite and Laponite^®^

**Figure 4:**
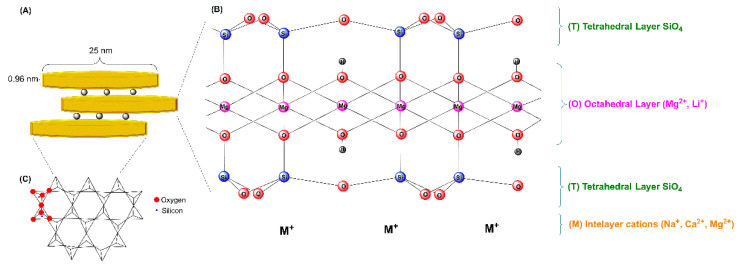
Schematic representation of the layered structure of hectorite-like clay. (**A**) Single hectorite nanocrystal geometry (disc-shaped), (**B**) atomic structure of hectorite-like clay, and (**C**) top view of the Si-O tetrahedral sheet.

Hectorite is a trioctahedral smectite with the formula (Si_8.0_)[Mg_6.0−x_Li_x_](OH.F)_4_O_20_M^n+^_xn_∙*m*H_2_O (M = Na, Li, or NH_4_). Figure 4 shows the structure of hectorite consisting of two sheets of tetrahedral silica with a central octahedral sheet containing magnesium (Si-O-Mg(Li)-O-Si). Each layer, 0.926 nm thick, consists of two octahedral sheets sandwiching an octahedral layer. Moreover, these layers have positive charges on the edges, while the basal faces have negative charges [40]. The layers are separated by hydrated cations (Na^+^ and Li^+^, among others) in the interlayer space. The amphoteric groups (such as Mg-O, Li-O, or MgO-Li) can be protonated or deprotonated depending on the pH.

Hectorite can be obtained from natural sources. However, it must be synthesized to work with a highly pure and reproducible material. Commonly, the number of moles of reactants to produce synthetic hectorite follow the ideal composition of natural hectorite. The synthesis conditions can control the structure and properties of synthetic hectorites. The methods of preparation include hydrothermal treatments, microwave-assisted, and melt synthesis, among others.

The hydrothermal reaction is the most common method for the synthesis of hectorite-like solids. This method was introduced by Strese & Hoffman [41]. The experimental procedure involves the reaction of oxides and hydroxides under basic pH at high temperature and pressure. Vicente et al. [42] reported a fast method to synthesize hectorite by a microwave hydrothermal method, proposing the use of brucite sheets as the crystallization nuclei of hectorite. Kalo et al. [43] reported the large-scale synthesis of sodium fluorohectorite (Na_0.6_[Mg_2.4_Li_0.6_]Si_4_O_10_F_2_) from the melt in an unsealed glassy carbon crucible at 1265 °C. Daab et al. [44] reported the synthesis of sodium hectorite by melt synthesis followed by annealing for 6 weeks at 1045 °C, carrying out the procedure in a gas-tight molybdenum crucible.

Laponite^®^ is the term introduced by Laporte Industries to describe synthetic hectorite-like clay minerals. This term has been used since then as a synonym of synthetic hectorite particles. Therefore, Laponite^®^ (LAP) is the commercial synthetic hectorite. Laponite consists of highly pure nanoparticle dispersions of large surface area (>350 m^2^/g); basically circular platelets of approximately 25 nm in diameter and 1 nm of thickness, possessing a negative face charge and a weak positive rim charge (Figure 4) [45]. Several types of commercially available laponites have been used in the development of nanovaccines, including LAP WXFP, LAP RD, and LAP FN, among others. The main difference between them is their chemical composition [46]. For example, the chemical composition of laponite WXFP is [(Si_8_Mg_5.34_Li_0.66_)O_20_(OH)_4_]·Na_0.66_ (HEC), for Laponite RD is [(Si_8_Mg_5.55_Li_0.43_)O_18.45_(OH)_7.36_]·Na_0.73_ (LRD), and for Laponite FN is [(Si_8_Mg_4.17_Li_1.27_)F_1.7_ O_18.01_(OH)_5.98_]·Na_0.54_ (LFN).

## 3. Functionalization of Nanoclays

To functionalize nanoclays toward developing nanovaccines, the tools are those available for any other adsorbent material: passive and active adsorption. The former exploits the use of weak forces such as hydrophobic, electrostatic, and van der Waals interactions, while the latter involves the generation of stronger, covalent bonds. All these interactions must be established between the functional molecule (antigen, ligand, or adjuvant) and the surface of the nanoclays. For passive adsorption, the nanoclays surface is rarely modified, while for active adsorption, reactive moieties (more reactive groups at milder conditions) must be first placed on the nanoclays surface. Due to the composition of the nanoclays, silanization is extensively used when pursuing active adsorption. Most of the procedures used to generate nanoclay-based vaccine prototypes are established on passive adsorption due to its simplicity. To the best of our knowledge, active adsorption has not been pursued to generate nanovaccines based on nanoclays. It has been mainly followed to generate adsorbent materials. Despite this, active adsorption using procedures to generate adsorbents is described here to establish all the approaches that could be used to generate nanovaccines. Finally, this section focuses on the nanoclays most reported thus far to generate nanovaccine candidates: layered double hydroxides (LDH), halloysite nanotubes (HNT), and synthetic hectorite (Laponite^®^).

### 3.1. Passive Adsorption

Nanomaterials functionalized with proteins constitute the basis of modern agglutination tests, lateral flow assays, targeted drug delivery, and tissue labeling techniques, among others. These are based on passive adsorption, a method originally reported to affix antibodies on latex microspheres for serological diagnosis [47], and on gold nanoparticles for immunolabeling [48], allowing them to directly reveal antigens in serums and on cells and tissues, respectively. Continuing this field, the indirect detection of antibodies followed afterward, as protein antigens can instead be passively adsorbed on nano- and micromaterials [49]. More advances and applications have resulted since then.

Passive adsorption started as a procedure to improve the stability of intrinsically hydrophobic sols (i.e., colloidal suspensions of solid particles) by combining them with hydrophilic substances such as proteins (e.g., albumin). This enabled the protection of ion-stabilized colloids (e.g., gold sols) that easily flocculate upon the addition of electrolytes [50] or the incorporation of insoluble and poorly soluble materials (e.g., orpiment, limonite) into aqueous suspensions [51]. Due to the low cost and simplicity of this method, passive adsorption is still extensively used. Moreover, its continued use and exploration in reliable immunodiagnostics can be applicable to the development of nanovaccines.

During passive adsorption, biomolecules freely bind the exposed surface of nanomaterials in suspension to form bioconjugates. This phenomenon occurs between the adsorbate (e.g., protein antigens) and the adsorbent (e.g., nanoclays) through intermolecular interactions (electrostatic, hydrophilic, hydrophobic, and van der Waals forces), which drive the adsorption process. Nanoclays with a certain net charge are stable in suspension due to electrostatic repulsion. As proteins have amino acid residues exposing positively and negatively charged groups, they adsorb on the surface of nanoclays of either charge once added to the same suspension. The adsorption of proteins on nanoclays is straightforward as both are simply incubated together during certain time.

To produce functional bioconjugates, specific concentrations of protein are usually tested against certain amounts of nanomaterial. In principle, the nanomaterial in suspension can be contacted with an excess of proteins in solution, to guarantee the complete functionalization of the nanoclay surface, while discarding unbound proteins afterward. In practice, the amount of protein necessary to produce stable bioconjugates is determined by exposing fixed amounts of nanomaterial to increasing concentrations of protein during certain time. In this case, the unbound proteins are quantified to estimate the minimum amount that must be adsorbed per amount of nanomaterial to produce bioconjugates that will not flocculate within the suspension. For protein antigens, finding a minimum required amount is important considering production costs and limitations.

Since nanoclays have hydroxyl groups on their surface, the adsorption of proteins primarily occurs through electrostatic and hydrophilic interactions. Nevertheless, this assertion disregards the different chemical structure of the edges, interlayer cations, and spacing within the nanoclays, leading to the underestimation of hydrophobic interactions [52]. As proteins are structured by enclosing their hydrophobic interior within their mostly hydrophilic exterior, their conformation would remain unaltered during their physisorption on hydrophilic surfaces, but it would involve conformational changes due to hydrophobic interactions, including protein-protein binding, protein clusters, and aggregates [53]. Moreover, the conversion between the different folded states that can exist in the protein will depend on physical alterations (temperature, mixing) or modifications by a chemical agent (solvent, surfactant, ligand).

In addition to testing different protein/nanoclay mass ratios to produce adsorption isotherms, the most common approach to properly adsorb proteins onto nanoclays requires adding the nanoclay suspension drop by drop into the protein solution under strong, constant mixing. The speed and order of addition are relevant: rapidly adding the nanoclay suspension into the protein solution, or adding the protein solution into the nanoclay suspension, will cause the formation of aggregates. In contrast, and as an example, adding LDH dropwise into BSA solutions under vigorous stirring results in homogeneous, well-dispersed BSA-LDH suspensions [54]. The protein/nanoclay mass ratio influences the final size of the protein-nanoclay conjugate. At higher ratios, the size of the conjugate is only slightly larger than that of the unmodified nanoclay, as there is sufficient protein to fully cover it with a monolayer. At lower ratios, the conjugates increase in size as they aggregate due to the bridging effect caused by the scarce proteins now shared between the nanoclays [55]. After combining both materials, all the methods reviewed also concur on stirring the mixture continuously for thirty additional minutes before separating the free protein in the suspension from the protein-nanoclay conjugates.

The adsorption of antigens depends on the type of nanoclay. Mg-Al layered double hydroxides (LDH) and hectorite (HEC) have been the most studied. HEC is a phyllosilicate smectite clay that is negatively charged. LDH are hydrotalcite-type clays that are positively charged. While LDH can adsorb, by electrostatic attraction, negatively charged biomolecules (e.g., DNA and RNA; as they have phosphate groups in their nucleotides), both LDH and HEC can adsorb zwitterionic biomolecules, such as amino acids, peptides, and proteins. However, even as their charge magnitude is similar, their adsorption capacity is considerably different. In a study comparing HEC (77 nm, −41 mV) and LDH (115 nm, +36 mV) using the same recombinant protein antigen (intimin β), HEC adsorbed four times more protein than LDH [56]. Other than describing the evident: the positively charged groups of intimin β strongly interacting with the negatively charged surface of HEC, and vice versa for LDH, the explanation of the adsorption difference between both nanoclays considered that the protein has more amino groups than carboxylate groups available. Intimin β is an outer membrane protein in *E. coli* (pathogenic and enterohemorrhagic) for intimate adherence and full virulence in initial stages of infection. The full protein in *E. coli* O157:H7 has a MW of 101.8 kDa and 934 amino acid residues, of which 86 and 79 have positively (NH₃^⁺^) and negatively (COO^−^) charged side chains, respectively. The recombinant, C-terminal domain of intimin β (Intβ) used in their work had an approximate MW of 46 kDa and 480 amino acids, with a comparable proportion of charged side chains as the complete protein. In a posterior study, with HEC (74 nm, −35 mV) and LDH (113 nm, +36 mV) and the same recombinant Intβ, they obtained similar results: HEC loading four times more Intβ than LDH, managing to notably reproduce the data for adsorption isotherms at the same equilibrium concentrations [57]. In a preceding study, they reproduced the same adsorption capacity and isotherm for Intβ with a similar HEC (73 nm, −33 mV), but in parallel with their following article no explanation is provided on the high adsorption capacity of HEC [46]. However, they reported the adsorption of Intβ with two other types of hectorite: LRD (30 nm, −19 mV) and LFN (155 nm, −55 mV), of smaller and larger size, and with less and more negative charge than HEC. With LFN, the adsorption increased 0.47 mg/mg over HEC (4.4 mg/mg), while the smaller LRD reached 2.4 mg/mg of Intβ/HEC. Considering the same weight of nanoclay, but now partitioned into the sizes reported, LRD should have a larger number of nanoclay particles available to adsorb more Intβ and increase its loading capacity. However, LRD barely adsorbed half the amount of Intβ compared to LFN. One reason could be the lesser negative charge of LRD attracting less Intβ, compared to HEC and LFN.

### 3.2. Active Adsorption

**Figure 5:**
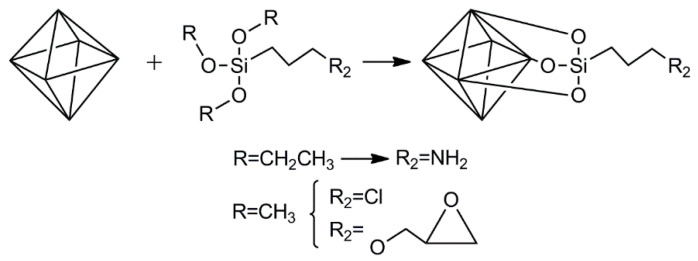
Three examples of organosilanes to modify the surface of nanoclays.

The chemical modification of nanoclays relies on the presence of hydroxyl groups on their surface. These hydroxyl groups could be used to attach other molecules using an intermediary. This intermediary group will change the surface such that a more reactive, convenient moiety exists. Due to the structure of nanoclays having SiOH groups (either by constitution or defects on the surface) or other OH containing groups, the obvious choice for this intermediary are organosilanes (Figure 5). These organosilanes are envisioned to react with the surface of the nanoclays, while presenting a chemical moiety that can be used to react with peptides or proteins. To the best of our knowledge, nanovaccines based on the chemical attachment of antigens on the surface of nanoclays has not been explored. Despite this, the chemical modification of nanoclays has been studied for other applications. In this section, some applications are briefly described and complemented by the procedures that could be used to actively adsorb antigens, ligands, and/or adjuvants on the surface of nanoclays. LDH, halloysite, and hectorite (Laponite^®^) are the main nanoclays that have been tested as nanovaccines; therefore, those are the ones covered in this section. Active adsorption of small molecules (such as peptides and ligands) on the surface of nanoclays must be pursued in the vaccinology field, as these molecules are expensive and cannot be used in large amounts (as in the case of passive adsorption using inexpensive proteins such as BSA). Moreover, even for large protein molecules, the use of recombinant proteins is limited for passive adsorption, as large amounts must be used to impart stability, which implies the generation of large amounts of recombinant protein (a situation which is not always possible). It is worth mentioning that once the nanoclays have amino or carboxylic groups (covalently grafted); they could be used to passively adsorb antigens (peptides or proteins) with opposite charge through electrostatic interactions.

#### 3.2.1. LDH

LDH naturally exist as hydrotalcite [58] or can be produced in the laboratory. The layer structure of LDH is derived from brucite (Mg(OH)_2_) upon substitution of some magnesium ions by trivalent ions (e.g., Al^3+^) [59]. Due to the presence of a high density of OH groups on the surface of LDH, the use of organosilanes to functionalize the surface has been widely reported.

Ádok-Sipiczki et al. [60] followed an approach to functionalize LDH that could be used to develop nanovaccines based on peptides or proteins as antigens. They covalently attached single-stranded DNA to Mg:Al or Zn:Al LDH using APTES as linker. Both LDH were produced upon the co-precipitation of the respective nitrate salts, followed by hydrothermal treatment. For functionalization, LDH were first calcined to produce LDO (layered double oxides). Afterward, these LDO were suspended in ethanol containing ammonium hydroxide, wherein an ethanolic solution of APTES was then added dropwise under stirring. The silanization reaction proceeded for 6 h. The aminated LDH were reacted with a nucleic acid strand containing a carboxylic group using carbodiimide chemistry with EDC/NHS (1-ethyl-3-[3-dimethylaminopropyl] carbodiimide hydrochloride/N-hydroxysuccinimide). In a similar initial procedure, a reflux condition was applied to attach APTES to the surface of Ca:Fe LDH with intercalated tartrate [61] or citrate [62]. Since peptides and proteins contain at least one carboxylic group in their structure, the approach followed by Ádok-Sipiczki et al. [60] could be immediately applied to produce nanovaccines with grafted antigens.

**Figure 6:**
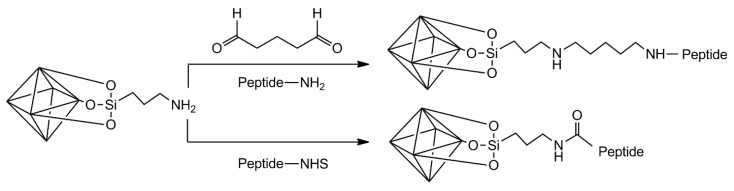
Two approaches to graft peptides or proteins to the surface of aminated LDH.

Once the surface of LDH has been grafted with amino groups from the silanization with APTES, this amino group can be used for the attachment of peptides or proteins. One option has been already mentioned and consists of attaching activated peptides or proteins (with NHS moieties) to this amino group. Another approach that could be followed relies on reductive amination processes, where a functional dialdehyde such as glutaraldehyde functions as linker between amino groups from LDH and amino groups from a peptide or protein (Figure 6). A one-step or two-step approach can be followed for this purpose. In the former, glutaraldehyde is added to a suspension of aminated LDH containing peptide or protein dissolved, while in the latter the surface or the aminated LDH is first reacted with an excess of glutaraldehyde. After removing unreacted glutaraldehyde, the surface of LDH now contains aldehyde groups that can be reacted with a peptide or protein. Crosslinking of the peptide or protein with glutaraldehyde is, therefore, reduced in the latter approach.

#### 3.2.2. Halloysite

For the development of nanovaccines with a grafted antigen, the antigen molecule should be located ideally on the outer surface of halloysite (lateral and edges). If the antigen is a whole protein molecule, its large size would restrict its entrance in the lumen of HNT (when a protein enters and reacts with active groups, the restriction will further increase). For the case of small peptides as antigen molecules, they could enter the lumen with fewer restrictions and become grafted in the inner surface. Therefore, the outer surface of the HNT should be reactive enough to guarantee successful grafting of peptides or proteins. The outer surface is composed of siloxane groups [25]. Si-OH groups exist on the edges of the HNT and in defects on the outer surface. To increase the reactivity of the outer surface toward organosilane molecules, several authors have activated this surface using acids, bases, hydrogen peroxide, or combinations, to favor the generation of a hydroxylated surface [63,64,65,66,67].

**Figure 7:**
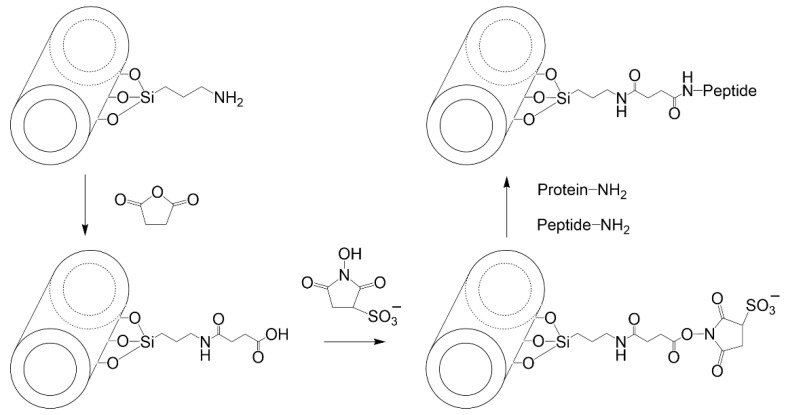
Grafting a protein or peptide on the surface of aminated HNT using succinic anhydride and carbodiimide chemistry.

Prinz Setter et al. [68] studied HNT with antibodies targeting bacteria. The approach followed could be easily translated to the generation of nanovaccines based on HNT. For this, HNT were first acid etched using sulfuric acid to roughen the surface of the native HNT, making it more reactive. Afterward, the surface of these HNT was silanized using APTES in the presence of dry toluene. Overnight reflux was applied to favor condensation. The authors then introduced carboxylic acid moieties by reacting the aminated HNT with succinic anhydride in DMF for 24 h at room temperature. The functionalization procedure continued by grafting protein A using carbodiimide chemistry (with EDC + sulfo-NHS). Finally, an antibody was added and allowed to interact with the grafted protein A. The procedure is depicted in Figure 7, showing how a peptide or protein molecule can be attached to HNT to develop nanovaccines.

The approach of aminating, carboxylating, and grafting amine-containing molecules on the surface of HNT through carbodiimide chemistry requires the use of expensive solvents such as DMF, expensive buffers such as MES, and easily hydrolysable compounds (EDC). A simpler approach to generate nanovaccines based on HNT would be the use of reductive amination chemistry. Once the surface of HNT is aminated, glutaraldehyde could be used to introduce aldehyde groups that can be later used to graft peptides or proteins as antigen molecules. A final reduction process with NaBH_4_ allows forming non-hydrolysable bonds [69].

#### 3.2.3. Hectorite (Laponite^®^)

**Figure 8:**
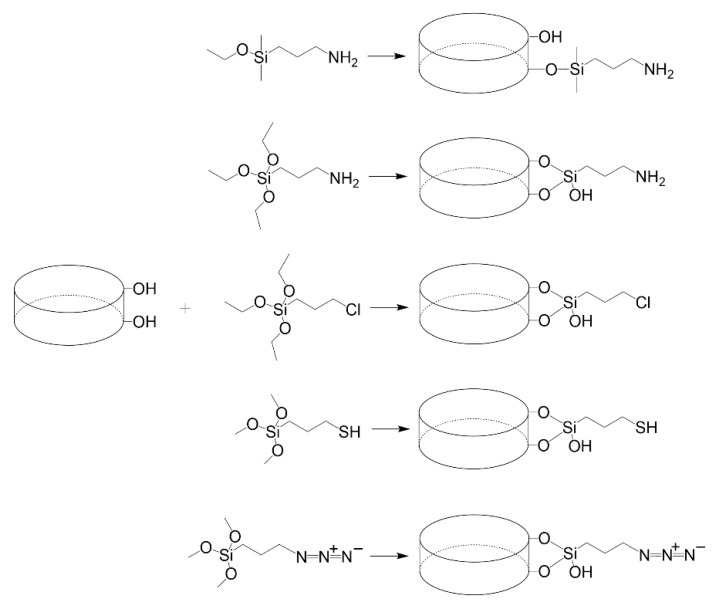
Mono-alkyl and tri-alkyl silanes reported to modify laponite.

Laponite^®^ is a synthetic silicate that is similar in structure and composition to hectorite [70]. Laponite is currently commercialized by BYK Altana, which offers several laponites with different constituents. Primary Laponite RD (LRD) is reported to be 25 nm across and 1 nm thick. These primary Laponite layers are usually stacked and separated by exchangeable cations [71]. The edges of Laponite have reactive -OH groups that can be used for silanization purposes [72]. Mono-alkoxy and tri-alkoxy silanes have been used (reported below), with the former generating a flat monolayer on the Laponite edge and the latter linking the sheets together [73]. All explored organosilanes to start functionalizing laponite for active adsorption of peptides or proteins is presented in Figure 8.

Mustafa et al. [74] grafted aminated dendrimers to Laponite for the loading and delivering of an anticancer drug. The approach followed could be easily implemented to attach a peptide or protein to the edges of Laponite. For this, 3-aminopropyldimethylethoxysilane (APES) was used to aminate the edges of Laponite in an aqueous medium. The amino groups were changed to carboxylic groups using succinic anhydride in DMSO. Finally, EDC+NHS were used to graft the aminated dendrimers. The aminated Laponite can also be used to react with NHS derivatives of peptides or proteins to generate nanovaccines. This approach was followed to attach fluorescamine or NHS ester of other dyes [75]. APTES has also been used to aminate the edges of Laponite, despite the risk of APTES crosslinking different laponite disks [76].

Laponite was grafted with molecules containing amino groups (melamine and biuret), using (3-chloropropyl) triethoxysilane (CPTES) as linker. For this, Gonzalez et al. [77] dispersed Laponite in water and CPTES was added. Afterward, the amino-containing molecules were added. This procedure can be adapted to attach peptides or proteins to the surface of Laponite. The use of CPTES to silanize Laponite was also used by Guerra et al. [78] to graft a thiol-containing molecule (which could be a synthetic peptide with an additional cysteine residue). Guimarães et al. [79] grafted 3-mercaptopropyltrimethoxysilane (MPTMS) to the surface of laponite to prepare an adsorbent for biomolecules. This thiolated Laponite could be used to attach a synthetic peptide with an additional cysteine residue in the presence of cystine.

Click chemistry was used by Colletti et al. [80] to generate Laponite triazole derivatives using propargyl alcohol. For this, 3-azidopropyltrimethoxysilane (AzPTMS) was mixed with Laponite in toluene under microwave irradiation. The azido-functionalized laponite was then contacted with propargyl alcohol, copper sulfate, and sodium carbonate. To attach a protein or peptide under a similar procedure, they must first be modified with 4-pentynoic acid using carbodiimide chemistry. Afterward, the resulting product (alkynyl labeled protein) can react with the azido-functionalized Laponite, using an aqueous environment with longer reaction times at room temperature or colder [81].

## 4. Nanovaccine Candidates Based on Nanoclays

This section describes nanovaccine candidates, where the system is mainly composed of nanoclays and adsorbed antigens (or other molecules), reported in the literature. This section also includes nanosystems where a coating for protection or stabilization has been applied. Table 1 summarizes the main immunological results of these nanovaccines. More complex systems, where nanoclays are used among other constituents in the final formulation, are described later.

Li et al. [82] prepared an anti-melanoma DNA vaccine based on LDH. For this, LDH were prepared by coprecipitation under an N_2_ atmosphere. The supercoiled pcDNA3-OVA plasmid was subsequently adsorbed on these LDH to create the nanohybrid DNA-LDH(R1), which had a size of 80–100 nm and a ζ potential of +19 mV. Using agarose gel electrophoresis, they found that the synthetic nanoclays were able to protect DNA from degradation upon treatment with DNase I enzymes. C57BL/6 mice were intradermally (i.d.) immunized with pcDNA3-OVA/LDH(R1), pcDNA3-OVA complexes, or pcDNA3-OVA/LDH(R1) in combination with CpG twice at a 1-week interval. Mice were subjected to a B16-OVA challenge one week after the immunizations. Immunization of mice with pcDNA3-OVA/LDH(R1) and pcDNA3-OVA/LDH(R1)+CpG significantly induced the delay of tumor growth and increased mice survival by 63 and 75%, respectively, in comparison with pcDNA3-OVA. In addition, the therapeutic effect was evaluated in mice with pre-established tumors. Three immunizations were administered at 4-day intervals forming the same groups previously described. The results showed tumor growth inhibition and an increase in mice survival from 25 days (pcDNA3-OVA group) to 40 or 45 days for the pcDNA3-OVA/LDH(R1) and pcDNA3-OVA/LDH(R1)+CpG groups, respectively. Significantly higher induction of humoral immune response was observed in terms of IgG antibody levels in mice immunized with LDH when compared to the pcDNA3-OVA or pcDNA3 groups. Interestingly, high IgG2a and moderate IgG1 levels were observed in mice immunized with LDH. In addition, high proliferative OVA-specific Th1 cell and CTL response in conjunction with IFN-γ production were observed in the LDH group when compared to the pcDNA3 and pcDNA3-OVA groups.

Yan et al. tested if LDH administered with the toll-like receptor (TLR) ligand CpG could modulate the immune response [83]. Ovalbumin (OVA) was used as the model antigen. LDH consisted of magnesium and aluminum, and were produced by coprecipitation and hydrothermal treatment. OVA was passively adsorbed to LDH with a maximum adsorption capacity of 0.577 mg/mg OVA/LDH, which was estimated using the Langmuir model. C57BL/6 mice were s.c. immunized with OVA, LDH, CpG + OVA (1:12 ratio), LDH + OVA (8:1 ratio), LDH + OVA (4:1), LDH + CpG + OVA (96:1:12 ratio), ALUM + OVA (8:1 ratio), and ALUM + CpG + OVA (96:1:12 ratio). At day 35, mice were challenged against B16/F10/OVA cells. LDH + OVA (8:1 ratio), LDH+OVA (4:1), and LDH + CpG + OVA induced higher IgG1 levels than OVA alone, and comparable antibodies levels with ALUM+OVA (8:1 ratio) at day 35. Interestingly, LDH + CpG + OVA showed higher IgG1 levels than CpG + OVA and OVA alone. At days 21 and 35, LDH + CpG + OVA induced the highest IgG2a levels than all other groups (e.g., an 8-fold increase was observed when compared to ALUM + CpG + OVA). These results suggested that LDH is an immunomodulatory adjuvant, capable of inducing both Th2 and Th1 cell responses. After the challenge, a tendency to delay tumor growth while incrementing survival was observed in the LDH + CpG + OVA group when compared to the OVA alone group (no statistical difference was observed) at 24 and 26 days. Comparable results were observed until day 42, only one mouse from the CpG-OVA and LDH-CpG-OVA groups had not been euthanized, this response can be elicited by CD8^+^ CTL infiltration in melanoma tissues. Interestingly, the groups that received ALUM showed side effects such as inflammation and hair loss in the injection site.

Hartwig et al. [84] used halloysite nanotubes (HNT) and carbon nanotubes (MWCNT) as carriers of a recombinant protein (rLipL32) to produce vaccines against leptospirosis. rLipL32 was physically adsorbed to the nanosystems for 24 h. Immunization protocols were designed to elucidate the immunogenic properties of the candidate vaccines. Golden Syrian hamsters received two doses at days 1 and 14 using HNT, MWCNT, or Alhydrogel, with or without adsorbed rLip32. After priming and boosting, rLipL32-MWCNT and rLipL32-HNT induced a significantly higher IgG response when compared to the negative control groups. Fourteen dpi rLipL32-HNT only induced a higher IgG response than rLipL32-Alhydrogel (positive control group). However, 28 dpi rLipL32-MWCNT and rLipL32-HNT induced a significantly higher IgG response when compared to the positive control group. No protective immunity was provided against a challenge with *Leptospira interrogans*. HNT can be functional as nanocarriers of antigens; however, it is necessary to evaluate case per case for other candidate antigens.

**Figure 9:**
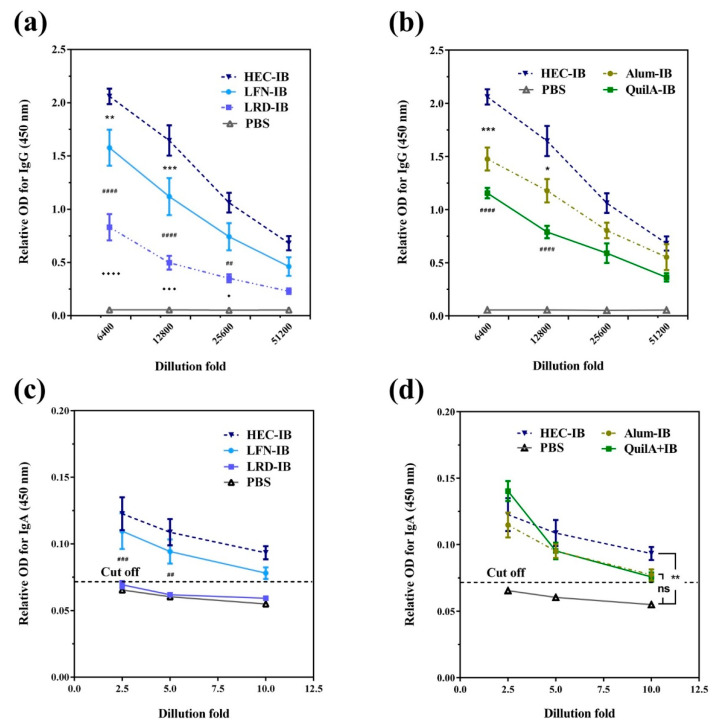
(**a**) Anti-IB IgG levels in sera from C57BL/6 J mice immunized with laponites LRD, HEC, and LFN), (**b**) Anti-IB IgG levels induced by HEC loaded with IB and its comparison with commercial adjuvants (Alum and QuilA) and PBS, (**c**) specific anti-IB SIgA in feces at day 49 for (**a**,**d**) specific anti-IB SIgA in feces at day 49 for (**b**). Symbols (*/♦/#) in (**a**,**c**) indicate differences between adjacent two groups, while symbols (*/#) in (**b**) indicate differences between the test laponite and commercial adjuvants (*Alum #QuilA). The cutoff was calculated as: Cut-off  =  mean  +  10 × SD. Data are expressed as mean  ±  S.E.M. (*n*  =  5). */♦ *p*  <  0.05; **/## *p*  <  0.01; ***/♦♦♦/### *p*  <  0.001; ♦♦♦♦/#### *p*  <  0.0001; and n.s. = non-significant [46].

Chen et al. [56] evaluated a vaccine against intimin β (IB) of diarrheagenic *E. coli* based on clay nanoparticles (LDH and hectorite). LDH were prepared by coprecipitation followed by hydrothermal treatment, while Laponite^®^ WXFP (HEC) was a commercial, synthetic hectorite. Physisorption was used to immobilize recombinant IB on the clay nanoparticles studied. The adsorption of IB on both materials followed a Langmuir isotherm, with HEC achieving a higher adsorption of IB (4.4 mg/mg) at equilibrium when compared to LDH (1.1 mg/mg). The immunization protocol was developed, immunizing C57BL/6J mice subcutaneously (s.c.) at days 1 and 21 with LDH (115, 243, or 635 nm) or HEC (77 nm) and IB (8:1 ratio) or QuilA and IB (1:1 ratio). Prime and boost s.c. immunization induced anti-IB IgG levels comparable to those induced by QuilA at a high dose (10 µg). At the low dose scheme (5 µg), the IgG levels in the QuilA group decreased, while this situation did not occur with the LHD and HEC groups (interestingly, these IgG levels were maintained up to 120 days). The Th2 immune response seemed to be more polarized at day 120 for the LDH and HEC groups; in addition, higher humoral immunity was recorded for LDH-IB. Synthetic hectorite was further evaluated by Chen et al. [46] using the same antigen and comparing three synthetic hectorites with different chemical composition: Laponite WXFP (HEC), Laponite RD (LRD), and Laponite FN (LFN). The antigen was physisorbed. C57BL/6J mice were s.c. immunized at days 1 and 21 with PBS (as negative control), Alum-IB and QuilA-IB (as positive controls, 1:1 ratio), and IB adsorbed on HEC, LFN, LRD, or LDH (32:1 ratio). As reported by these authors, HEC-IB induced the strongest IgG immune response when compared to Alum and QuilA adjuvants, while promoting the strongest secretion of sIgA (Figure 9). Interestingly, HEC-IB can induce a cellular immune response, which was evidenced by the overexpression of IFN-y in splenocytes. An efficient sIgA response was observed with inhibition of EHEC O26 attachment to ruminant and human intestinal cells using in vitro assays. In addition, HEC showed greater efficiency than Alum and QuilA at inducing the maturation of RAW 264.7 macrophages mediated by CD86, with higher secreted levels of IFN-γ and IL-6. These works represent a promissory approach for diarrheic diseases caused by diarrheagenic *E. coli*.

Zhang et al. [85] determined if LDH could deliver molecules to dendritic cells. The molecules tested were tyrosinase-related protein 2 (Trp2) and indoleamine 2,3-dioxygenase siRNA (siIDO). LDH were prepared by coprecipitation and hydrothermal treatment. Both molecules tested were physically adsorbed to LDH, siIDO was adsorbed first, generating the intermediate LI, followed by Trp2 (maximum adsorption capacity of 0.4 mg/mg) to generate the candidate TLI. Most of the siIDO adsorbed was prevented from degradation upon contacting the nanosystem with RNase A, concluding that siIDO was primarily intercalated within the interlayers of LDH. First, the authors investigated the possible cytotoxicity of LDH using in vitro studies with BMDC (bone marrow-derived dendritic cells). They showed cell viability up to 80% using LDH at 200 μg/mL. They also evaluated the uptake, internalization, and distribution pathways in BMDC and DC2.4 cells, showing that TLI entered the cells through the endosome pathway. Afterward, they escaped from the endosomes and distributed in the cytoplasm and around the nucleus (LDH protected siIDO and Trp2 from degradation in endosomes). In BMDC, the authors demonstrated that TLI induced the reduction of IDO expression and conduced to IDO-mediated immune suppression. Using in vivo studies, they showed that siIDO mainly distributed in lung and liver, while TLI mainly distributed in lung, liver, and the paracortex of lymph nodes, providing additional uptake opportunities by DC and T cells to induce effective immune responses. In addition, an immunization scheme was developed to evaluate the therapeutic capability of TLI. At day 0, C57BL/6 mice were inoculated with 2 × 10^5^ B16-F10 melanoma cells, and at day 4 mice were immunized with LDH, Trp2, LDH-siIDO (LI, at 5:0.3 ratio), LDH-Trp2 (TL, at 5:1 ratio), and LDH-siIDO-Trp2 (TLI, 5:0.3:1 ratio). TLI induced the inhibition of tumor growth better than the other groups, including the positive control groups. In addition, TLI induced a significantly higher CTL response than TL, while CD8^+^ T cells were capable of producing IFN-γ. This approach is promising for cancer therapy, which can represent significant advancement in the development of an effective and efficient vaccine against melanoma cancer.

Chen et al. [57] prepared LDH and HEC (Laponite WXFP) with adsorbed intimin β (IB) as biodegradable depots for sustained antigen stimulation. IB is an outer membrane adhesin of pathogenic *E. coli* [93]. IB was physisorbed to LDH and HEC. The authors established that the size of the LDH and HEC significantly increased when changing the dispersing medium from water to PBS, even in the presence of adsorbed IB. The size of the nanovaccines slightly increased (from 107 to 127 nm for LDH and 73 to 130 nm for HEC) in the presence of IB when using simulated medium (simulating the environment of subcutaneous injection) as the dispersing agent. C57BL/6J mice were s.c. immunized on days 0 and 21 to evaluate the adjuvant capacity of LDH and HEC loaded with IB; PBS was used as vehicle and negative control while QuilA-IB and Alum-IB were used as positive controls. The formulations were administered using a 32:1 ratio (only QuilA-IB was administered at a 1:1 ratio). Following immunization, potent cellular and humoral immune responses were induced in groups immunized with LDH-IB or HEC-IB when compared to the positive control groups (QuilA and Alum), evidenced in terms of IgG response and splenocytes secreting IFN-γ. They observed the formation of nodules with a loose structure at the injection site in groups immunized with LDH-IB or HEC-IB. At day 35, the cells recruited were mainly leukocytes and macrophages. These results evidenced the capability of nanoadjuvants to induce long-lasting immune responses. In addition, the antigen remaining in these nodules was 51.6 and 71.5% for LDH and HEC, respectively, suggesting that nanoclays allowed sustained antigen release. Overall, the candidate vaccines induced the formation of depots that were readily biodegradable, resulting in sustained release and recruitment of immune cells, with the induction of humoral immune memory T-cell responses. As this work showed an adequate approach, long-term humoral immune responses can be further evaluated.

In a related work, Chen et al. [86] used LDH an HEC as nanoadjuvants of three recombinant antigens: IB (intimin β) and two proprietary antigens (Pag1 and Pag2) to generate a multivalent nanovaccine against pathogenic *E. coli*. LDH consisted of Mg and Al and were prepared by coprecipitation followed by a hydrothermal treatment. Laponite WXFP (HEC) was a commercial, synthetic hectorite. The maximum antigen adsorption capacities using LDH were 1.10, 0.28, and 0.42 mg/mg for IB, Pag1, and Pag2, while those for HEC were 4.18, 1.77, and 3.88 mg/mg, respectively. The three antigens were simultaneously adsorbed onto LDH or HEC. Female C57BL/6J mice were s.c. immunized with IB, Pag1, and Pag2 adsorbed on QuilA (1:1 ratio for each antigen) and one, two, or three antigens (Pag1, IB or Pag2) adsorbed on LDH or HEC (32:1 ratio for each antigen) at days 1 and 21 with sacrifice at day 108. After prime and boost s.c. immunization, the induction of IgG antigen-specific antibodies was more efficient in groups receiving LDH or HEC with the three antigens when compared to single and double antigens. Interestingly, HEC induced greater levels of antibodies than LDH. In addition, sIgA antigen-specific increased at day 108. This humoral immune response is greater and more durable than that observed in the positive control group (using QuilA). Interestingly, a more efficient cellular immune response was induced in splenocytes from mice immunized with HEC-IB-Pag1-Pag2 or LDH-IB-Pag1-Pag2 stimulated simultaneously with the three antigens when compared to individual stimulus. The inclusion of multiple antigens in the same formulation has only been evaluated in this work, resulting in interesting synergic effects.

**Figure 10:**
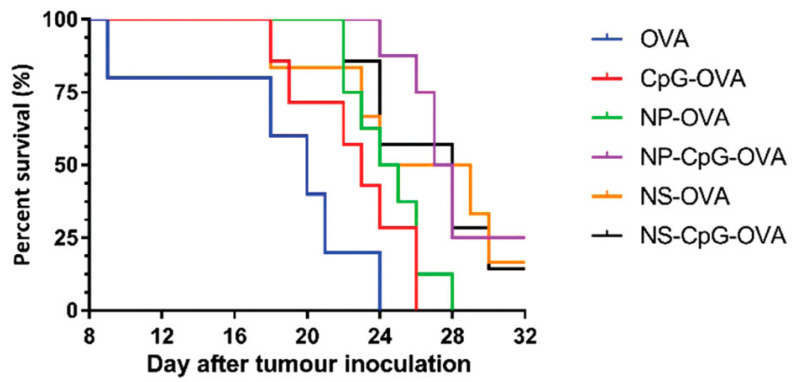
Percent survival of immunized mice challenged with EG7-OVA lymphoma cells (*n* = 8 per group) [87].

Yan et al. [87] tested LHD nanoparticles (NP) and nanosheets (NS) as components of anti-tumor vaccines using ovalbumin (OVA) as the antigen. NP were prepared by coprecipitation followed by hydrothermal treatment, while for NS the coprecipitation occurred in the presence of lactic acid, and gentle sonication was applied before a hydrothermal treatment. NP and NS were loaded with OVA using passive adsorption. C57BL/6 mice were s.c. immunized using a prime and boost protocol at a 2-week interval. Following immunization, higher production of IgG1 was induced in the groups that received the nanocarriers than the control groups. The groups that received NS-CpG-OVA or NP-CpG-OVA showed a higher humoral immune response in terms of IgG1 production when compared to not using nanocarriers, and the IgG1 levels were maintained until day 42. Interestingly, a prominent induction of humoral immune response was observed in the NS-OVA and NS-CpG-OVA groups. This indicate that NS have better adjuvant properties than NP. Comparable results were observed in the induction of IgG2a antibodies, as the production of these antibodies increased from day 29 after the first immunization. The IgG2a/IgG1 ratio indicates that the candidate vaccines NP-CpG-OVA and NS-CpG-OVA induced a Th1 immune response. The cellular immune response was evaluated in spleens isolated from immunized mice using an ELISPOT assay. At day 22, the splenocytes from the NP-CpG-OVA and NS-CpG-OVA groups showed a higher cellular response (up to 4.6-fold when compared to NP-OVA and NS-OVA), the quantities of IFN-y producer splenocytes increased 3.5-fold when compared to CpG-OVA. The synergic effect between CpG and NS or NP was observed in the induction of a CTL specific immune response in the presence of the SIINFEKL peptide (ovalbumin H-2Kb-restricted CTL epitope). Interestingly, at day 57, splenocytes from NP-CpG-OVA and NS-CpG-OVA showed a higher cellular response than all other groups. Half of the immunized mice were challenged with EG7-OVA tumor cells. The NP-CpG-OVA or NS-CpG-OVA groups were capable of inducing a reduction in tumor growth, while extending mice survival (Figure 10). This approach describes the potential of LDH-NP for their use as delivery vehicles in immunotherapies against cancer.

In an interesting work, Yan et al. [93] prepared LDH using coprecipitation and hydrothermal treatment and used them to elucidate the pathway by which these nanoparticles promoted immune responses. RAW 264.7 and BMDC cells were used to evaluate the cellular uptake of LDH-FITC (LDH-fluorescein isothiocyanate); this uptake was time and dose dependent. Additionally, they observed faster uptake at the first 4 h than the last 4 h evaluated. On the other hand, the release of this internalized LDH-FITC was evaluated. However, a passage of LDH to next cell generations was observed instead of cell exocytosis. Interestingly, the intercellular exchange was evidenced in RAW 264.7 using two different LDH complexes, LDH-FITC and LDH-CR, and LDH-Congo red. The results suggested that macrophages exchanged the internalized LDH complex with each other. In addition, the maturation of BMDC was evaluated using an LDH-OVA complex (2:1 ratio). The results showed significantly higher maturation of the MHC II DC population stimulated with LDH-OVA in comparison with OVA alone or the control group. The antigen presentation was evaluated in DC 2.4 cells stimulated with LDH-OVA, the 25-D1.16 antibody that specifically binds to the SIINFEKL/H-2κb complex was used to evidence the cross presentation through the MHC I pathway. Interestingly, LDH-OVA highly promoted the antigen complex presentation on the DC surface than OVA alone or medium control. Finally, B3Z cells (CD8+ T-cell hybridoma) that can recognize the SIINFEKL/H-2κb complex presented by DC were used to confirm the cross presentation. These authors confirmed more efficient cross presentation by DC pulsed with LDH-OVA than the control groups. Altogether these results provide new insights in the adjuvant mechanism of LDH, which could help in the design and development of new nanovaccine candidates.

Yu et al. [94] adsorbed BSA on LDH that were later coated with chitosan and alginate to generate nanocomposites for enhanced oral vaccine delivery. LDH were synthesized using co-precipitation, followed by hydrothermal treatment. LDH were contacted with BSA for passive adsorption. These LDH@BSA were coated with chitosan (CHT that was crosslinked with tripolyphosphate) and alginate (ALG that was crosslinked with CaCl_2_) to generate ALG-CHT-LDH@BSA. BSA-FITC was used to determine the profile release by changing pH every 2 h, starting at 1.5 and changing to 6.8 and 7.4. BSA was completely released at pH 1.5 within 1 h when only using LDH. For the complete system (ALG-CHT-LDH), 40% of BSA was released within 15 min at pH 1.5. This percentage slightly increased when changing pH to 6.8 and 7.4. The authors evaluated the cellular uptake of BSA-FTIC, LDH@BSA-FITC, and CHT-LDH@BSA-FITC in colon carcinoma (Caco-2), HT 29 and RAW 264.7 macrophage cell lines. High uptake was observed in all cell lines that were stimulated with CHT-LDH@BSA-FITC. In addition, this uptake was time and dose dependent. The CHT-LDH@BSA-FITC system improved internalization in intestinal and macrophage cells, mainly. However, immunization studies are necessary to evidence the adjuvant capability and efficacy of this candidate vaccine.

**Figure 11:**
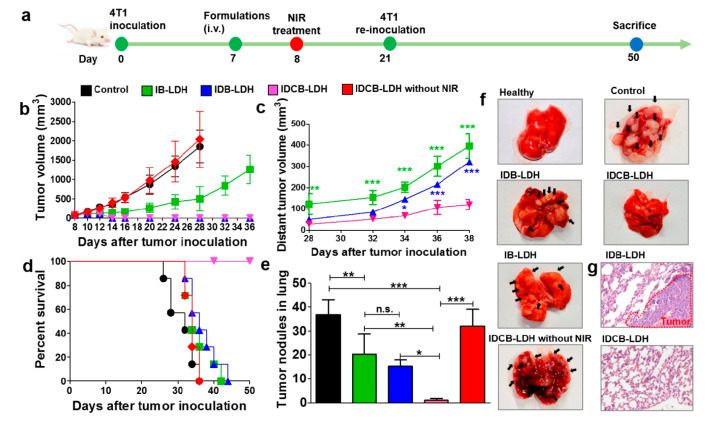
(**a**) Timeframe to evaluate the formation of distant tumors (modeling and treatment), (**b**) and (**c**) volume change of primary and distant tumors, respectively; (**d**) mouse survival for the different treatments, (**e**) average tumor nodules in the lungs of immunized mice, (**f**) dissected lungs of immunized mice, and (**g**) H&E staining of lung sections for IDB-LDH and IDCB-LDH treated mice. * *p* < 0.05, ** *p* < 0.01, *** *p* < 0.001, n.s. = non-significant [95].

Zhang et al. [95] developed a multifunctional nanomedicine based on LDH that combined photothermal therapy, chemotherapy, and immunotherapy using indocyanine green (ICG), doxorubicin (DOX), and CpG, respectively. CpG (cytosine-guanine oligodeoxynucleotide) is a TLR9 (toll-like receptor 9) agonist that stimulates anti-tumor immunity [96]. To prepare this multifunctional nanosystem (IDCB-LDH), LDH were first adsorbed with BSA, followed by ICG. After washing and redispersion, the nanoparticles were contacted with DOX (added as DOX/DNA pro-drug), followed by CpG. DNA is used as linker to adsorb DOX and attach to the positively charged LDH [97]. Cellular uptake and cytotoxicity to 4T1 and MCF-7 cancer cell lines were evaluated using DOX, DOX/DNA, ICB-LDH (without DOX), and IDCB-LDH as stimuli, NIR irradiation were applied in some wells of the culture plates. IDCB-LDH were highly internalized (up to 79.5%) when compared to DOX/DNA (66.5%), IDCB-LDH were colocalized at endosomes/lysosomes followed by IDCB delivery into the cytosol. NIR intensified this internalization and delivery. On the other hand, IDCB-LDH induced higher cell death than ICB-LDH. This observation can be explained by the efficient cell uptake and internalization; noteworthy, these phenomena were potentiated when NIR irradiation was applied. BALB/c mice with established tumor (100 mm^3^ and induced with 4T1 cells) were i.v. immunized with saline, IB-LDH (without DOX and CpG), IDB-LDH (without CpG), and IDCB-LDH; 16 h post-injection, the mice were treated with NIR irradiation (an additional group was immunized with IDCB-LDH and no NIR irradiation was applied). The skin temperature from the IDCB-LDH group after NIR irradiation was higher (from 32 to 48 °C) than the control group, the tumor volume decreased and almost disappeared in the mice from this group, and no changes were observed in the IDCB-LDH without irradiation and control groups. To evaluate the formation of distant tumors, the mice group that received IDCB-LDH was inoculated with 4T1 cells on day 21 at a different site. Interestingly, the development of distant tumors was inhibited in this group (80% of reduction). Moreover, mice survived 50 days following the inoculation of tumors (Figure 11). The metastasis in all groups was evaluated in lungs at day 28, while no metastasis was observed in the IDCB-LDH group. However, in all other groups many tumor nodules were observed. In the IDB-LDH group, all mice died at day 44, despite the reduction of the primary tumor. IDCB-LDH induced BMDC maturation (CD40+ CD80+ CD86+) and activation of more T CD8^+^ IFN-γ^+^ and CD4^+^ IL-4^+^ cells. In addition, higher levels of IFN-γ and IL-4 were secreted by splenocytes from mice treated with NIR than those without NIR. In another approach, BALB/c mice were inoculated with 4T1 cells in two sides of the lower back on day 0, followed by immunization at day 5 with the formulations previously described and irradiation of one side of the lower back. High levels of inflammatory cytokines were detected in serum (IL-6, IL-12, and TNF-α) from mice immunized with IDCB-LDH and NIR irradiation. Draining lymph nodes (dLN) showed higher activated DC from mice immunized with IDCB-LDH and NIR irradiation than all other groups. In addition, more CTL were recruited in distant tumors, while less Treg cells were observed. In summary, CpG served as an adjuvant that induces antitumor responses, which can efficiently prevent tumor recurrence, lung metastasis, and distant tumor growth. Moreover, the combination with ICG and DOX/DNA improved the efficacy of this candidate vaccine, resulting in synergistic and specific therapy to cancer cells that could offer a promissory approach in cancer treatment.

Oliveira et al. [88] developed a DNA vaccine to protect hamsters against Leptospira infection using HNT and multi-walled carbon nanotubes (MWCNT). Only the results for HNT are highlighted here. A plasmid (pTARGET/lemA) was produced in *E. coli* that codes for the LemA antigen. LemA is a putative lipoprotein with an M3 epitope conserved in pathogenic *Leptospira* spp. [98]. The authors described the evaluation of toxic properties through cytotoxicity assays in the Chinese hamster ovary (CHO) cell line, reporting no toxic effects in cell growth at concentrations up to 50 µg/mL. Afterward, they evaluated the efficiency of the vaccine to deliver DNA in the same cell line using HNT and MWCNT. Interestingly, a more efficient delivery (similar to the positive control) was obtained by MWCNT plus pTARGET/lemA, followed by HNT plus pTARGET/lemA. Following i.m. immunization on days 0 and 21, high levels of IgG antibodies were induced in mice receiving MWCNT plus pTARGET/lemA. Similar antibody levels were observed in the HNT plus pTARGET/lemA mice group. Interestingly, IgG antibodies levels were maintained until day 42. Hamsters were challenged with *L. interrogans* at day 42, revealing protective immune responses of 83 and 66% for MWCNT plus pTARGET/lemA and HNT plus pTARGET/lemA, respectively. This study demonstrates the potential of nanoclays as delivery agents within a leptospirosis vaccine.

Wu et al. [89] used LDH as adjuvants for an inactivated vaccine in pigs, composed of inactivated foot-and-mouth disease virus (FMDV). LDH were prepared by coprecipitation and hydrothermal treatment. FMDV was adsorbed on LDH using varying concentrations of virus with a fixed amount of LDH. The maximum loading ranged from 0.16–0.31 µg/µg FMDV/LDH. Centrifugation allowed recovery of the vaccine. First, the authors evaluated the possible cytotoxicity of LDH in BHK-21, MDBK, and SKC cells. No toxic effects were observed in concentrations of LDH up to 40 μg/mL. In a one-dose s.c. immunization scheme, inactivated FMDV was administered together with Montanide ISA-206 (M-ISA-206), LDH (1:1 ratio), or alone to BALB/c mice. Mice immunized with LDH+FMDV induced production of IFN-γ at day 14 comparable with the group immunized with M ISA-206; interestingly, the anti-FMDV antibodies levels were higher with LDH when compared to M-ISA-206 from day 56 post-immunization, and these antibody levels were maintained until day 98. On the other hand, pigs were i.m. immunized with PBS alone or inactivated virus O/MYA/BY/2010 together with M-ISA-206 or LDH (1:1 ratio). Similar levels of neutralizing antibodies were observed for LDH + FMDV and FMDV + ISA206, both higher than the PBS control group. Antibody levels decreased at day 28 and increased again at day 56 in the LDH group, indicating continuous antigen release by the clay. LDH can act as an adjuvant that induces both cellular and humoral immune responses with a sustained release of target antigen. In addition, the humoral responses are comparable to those attained with a commercial adjuvant.

**Figure 12:**
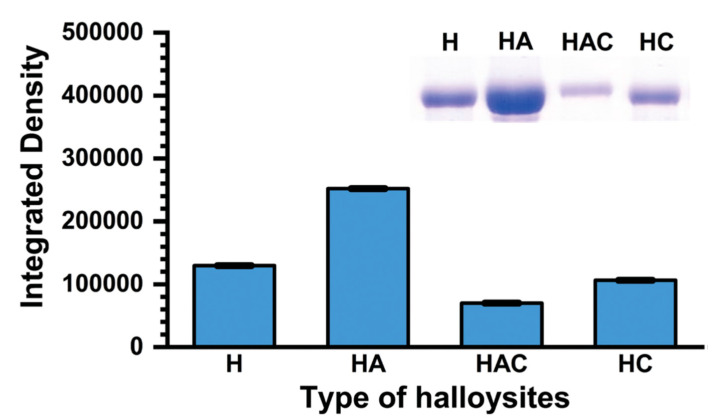
Densitometric analysis (from an SDS-PAGE analysis) of halloysites with adsorbed BSA. H, HC, HA, and HAC correspond to commercial HNT, HNT with chitosan physically adsorbed (HC), HNT with grafted APTES (HA), and HNT with grafted APTES (later modified with chitosan using glutaraldehyde as linker) (HAC), respectively [91].

Pumchan et al. [91] adsorbed BSA on natural and modified halloysite (HNT) to generate an oral biologics carrier. The modified halloysite included: halloysite with chitosan physically adsorbed (HC), halloysite with grafted APTES (HA), and halloysite with grafted APTES that was later modified with chitosan using glutaraldehyde as linker (HAC). The adsorption of BSA was simply performed by contacting natural or modified halloysite with a BSA solution (Figure 12). At pH 8, HA and HC immediately started to release BSA, while with HAC, the release started at 3 h. When contacting the materials with 0.3% bile salt solution, all materials started to release BSA by 0.5 h. The release followed the order: HC > HA > HAC. Additionally, these authors prepared a vaccine using a mixture of HNT and formalin-killed whole cells (FKC) of *Streptococcus agalactiae*. To evaluate the candidate vaccine as an oral delivery vehicle, Nile tilapia fish were immunized for three weeks with FKC loaded in HNT, sprayed in feed pellets. After three weeks, an antibody response was induced in 60% of the fish fed with HNT loaded with FKC, while for the control group no immune response was induced. The levels of humoral response in the HA-FKC and HC-FKC groups were time dependent. This work demonstrates the feasible use of HNT as delivery vehicles of antigens using an edible vaccine approach.

Zhang et al. [90] coloaded OVA and CpG onto LDH as an antitumor nanovaccine. Five different sizes of LDH were tested in the 77–285 nm range. All sizes were prepared by coprecipitation of aluminum and magnesium salts followed by different hydrothermal treatments. The adsorption of OVA was accomplished by adding (dropwise) an LDH suspension to an OVA solution (containing BSA) under stirring. CpG was subsequently added for adsorption to create CO-LDH. The size range of the nanovaccines increased to 137–438 nm after 24 h in FBS (fetal bovine serum). No OVA release was detected in FBS, while most of the OVA adsorbed was released after 2 h in an acidic (pH 4.5) medium. Cellular uptake efficiency and antigen presentation were evaluated in BMDC, the nanoclays were rapidly internalized and located in lysosomes, escaping from there in 4 h. CO-LDH-215 were the most efficient at delivering OVA in BMDC. In addition, the expression of H2kb/SIINFEKL was proportional to the size, CO-LDH-215 promoted antigen presentation more efficiently. One day after i.v. administration, CO-LDH-215 were captured in the spleen 24 h post-injection. C57BL/6 mice were immunized i.v. with the CO-LDH nanovaccines at days 0 and 7. At day 18, E.G7-OVA tumor cells were inoculated into mice. One week after the immunization scheme, the mice showed induction of humoral response, the production of IgG antibodies was size-dependent in terms of strength and polarization of the immune response. CO-LDH-215 induced the most potent anti-tumor humoral response. CO-LDH-215 and CO-LDH-106 induced Th1-type immune response, while CO-LDH-77 induced Th2 type immune response, which indicated that the size of nanoparticles influenced the polarization of the immune response. The candidate nanovaccines were tested in prophylactic studies where tumor induction occurred using the E.G7-OVA cell line. The results showed total protection by CO-LDH-106 and CO-LDH-215, in terms of immune response induction, mainly by IFN-γ producer cytotoxic lymphocytes (CTL). In addition, the antitumor therapeutic efficacy evaluated in two routes (i.v. and s.c.) revealed more efficient tumor inhibition (87%) for the i.v. immunization scheme in comparison with s.c. immunization (52%), due to the time-consuming and slow antigen presentation process. The i.v. administration of this kind of vaccine represents a novel approach with direct delivery of nanovaccines into the spleen resulting in enhanced immunotherapeutic efficacy.

## 5. Nanovaccine Candidates Based on Complex Formulations Containing Nanoclays

Wicklein et al. [15] reported the design and preparation of clay-lipid nanohybrid materials as adjuvants for influenza vaccines. The nanohybrids were prepared by adsorbing liposomes (prepared from phosphatidylcholine, PC) on sepiolite (SEP), and by co-precipitation of Mg/Al LDH in the presence of liposomes (this procedure generated LDH of 40–50 nm in diameter). Afterward, inactivated virions (A/PR/8/34-H1N1 strain) were contacted with the nanoclays to create the nanovaccine candidates SEP-PC and Mg/Al LDH-PC. Their thermostability was determined by haemagglutinin activity. The highest thermal stability was observed in viruses coupled to SEP-PC and Mg/Al LDH-PC in comparison with Al(OH)_3_. In addition, after lyophilizing the bioconjugates, the bioactivity of the virions was conserved. An immunization scheme was designed to evaluate the candidate vaccines and BALB/c mice were s.c. immunized at days 0 and 18 with 3 µg of virus. Sera samples obtained four weeks after the last immunization revealed high titers of specific antibodies in mice immunized with SEP-PC, similar to those observed in the group immunized with Al(OH)_3_. The major type of antibodies produced by this adjuvant were IgG1, indicating the induction of humoral response. Interestingly, SEP-PC induced higher IgG2a antibodies levels, indicating the induction of a Th1 immune response associated with cellular immune response capable of eliminating viral infections. These results demonstrate the potential of this kind of nanoclay as carriers for candidate vaccines.

Zhao et al. [99] synthesized core-shell structures to generate a DNA vaccine against Newcastle disease virus (NDV). The core consisted of silica nanoparticles, while the shell was composed of LDH. The plasmid DNA (pVAX1-F(o) DNA carrying the F gene of NDV) was loaded onto the silica nanoparticles aminated with APTES to create pFDNA-SiO_2_-NPs. LDH were afterward produced by coprecipitation in the presence of pFDNA-SiO_2_-NPs to generate the nanovaccine candidate pFDNA-LDH@SiO_2_-NPs. An in vitro DNA release study established that the plasmid DNA is slowly released in PBS, reaching a cumulative release of 91% after 288 h. The cytotoxicity of pFDNA-LDH@SiO_2_-NPs was evaluated in chicken embryo kidney cells. They did not observe changes in morphology, and a survival rate of 84% was achieved. Afterward, the safety of the candidate was assessed in SPF chickens i.m. and i.n. immunized with pFDNA-LDH@SiO_2_-NPs. The control group consisted of animals i.m. immunized with PBS. No abnormal behavior or water intake was observed. Moreover, the pathological section analysis revealed normal structures. To evaluate the induction of immune responses, chickens were i.m. immunized with PBS, LDH@SiO_2_-NPs, naked DNA, or pDNA-LDH@SiO_2_-NPs and i.n. with pDNA-LDH@SiO_2_-NPs, prime and boost schemes separated for two weeks were used. IgG titers significantly increased from the third week in groups receiving pDNA-LDH@SiO_2_-NPs by i.m. or i.n. routes. Interestingly, the antibodies titers of the i.n. group were maintained for five more weeks, the highest titers were reached at week 5 (up to 8.667 ± 0.577). Similar results were observed for IgA titers, the highest and sustained IgA titers in serum and other sites (Harderian glands, bile, and tracheal fluid) were observed in the group immunized with pDNA-LDH@SiO_2_-NPs. The cellular immune response was assessed using in vitro studies; they revealed higher stimulation of lymphocytes in the pDNA-LDH@SiO_2_-NPs group when compared to the other groups. Finally, chickens were subjected to a challenge with the virulent strain F48E9, obtaining a 100% protection. This candidate vaccine offers a promising approach for comfortable vaccines, capable of sustaining antigen release in a safe and efficient manner.

Lee et al. [100] reported the development of alginate beads with potential use as an oral vaccine using formalin-killed cells (FKC) from *Streptococcus parauberis*, a bacterial pathogen associated with epizootics streptococcosis infection in olive flounder [101]. FKC were encapsulated in polymeric beads of cross-linked alginate-Ca^2+^. The encapsulation efficiency and media-dependent release were controlled by pre-treatment of the FKC with kaolinite (KA) and layered double hydroxide (LDH) nanoparticles as colloidal state controllers. The encapsulation efficiency and loading capacity of the FKC in the alginate were found to be highly dependent on the clay nanoparticles used as ingredients. LDH seemed to reduce the encapsulation efficiency, while KA considerably increased it. This difference can be attributed to the difference in the surface charge. KA has an electric point between 4 and 6 [102], while LDH have a higher isoelectric point of 11 [103]. It is known that *S. parauberis* has an isoelectric point of ~4 [104], indicating a negative surface charge at neutral pH. Because of this, the authors suggested an electrostatic interaction between LDH particles and FKC that caused the formation of large agglomerates, affecting the encapsulation. On the other hand, the electrostatic repulsion between KA and FKC kept the former well dispersed, favoring the encapsulation efficiency. The FKC release was studied under two conditions, gastric solution (pH 1.2) and intestinal solution (pH 6.5). The release in deionized water (DW) was completed after 2 h in FKC alone-encapsulated alginate beads, due to their swelling. The presence of KA or LDH did not show measurable FKC release in the gastric solution within 4 h. The clay nanoparticles swell water instead of alginate, prohibiting the swelling of alginate beads and preserving the encapsulated FKC. On the other hand, the FKC release was considerable in the intestinal condition. This behavior was attributed to the alginate network disrupted by the clay nanoparticles; they were expected to make holes in the alginate matrix, causing a greater amount of released FKC. The best results were observed in the FKC-LDH system since LDH agglomerates in large lumps and a larger amount of FKC can be released. Immunologic assays were not conducted in this study.

**Figure 13:**
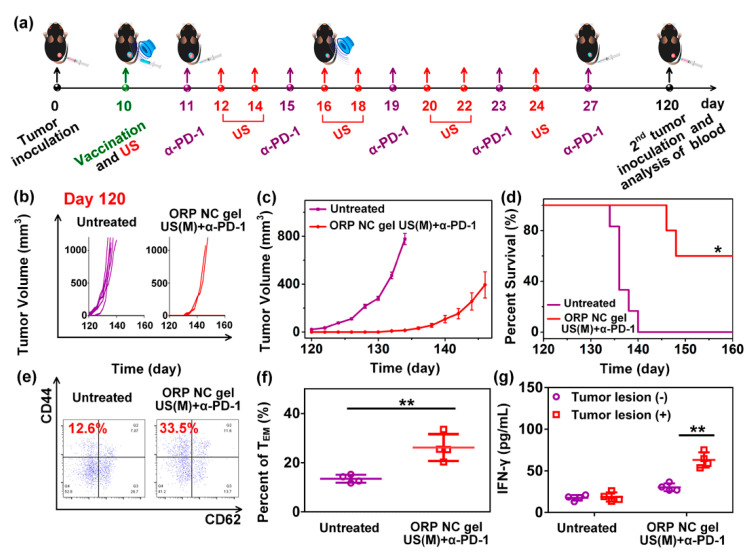
Long-term immune memory effect of the ORP NC gel. (**a**) The sequence of injections and ultrasound treatment, (**b**) individual and (**c**) average tumor growth curves mice with rechallenged tumors, (**d**) percent survival of mice after immunization with the treatments indicated in (**b**,**e**) representative flow cytometry plots and (**f**) statistic data (**f**), to show proportions of effector memory TEM in the peripheral blood at day 120, and (**g**) IFN-γ secretion from restimulated peripheral blood lymphocytes collected on day 120. Growth curves represent mean ± SEM. Survival curves were obtained using the Kaplan−Meier method. Data are presented as mean ± SD (*n* = 4). * *p* < 0.05, ** *p* < 0.01 [105].

Meng et al. [105] developed an easy-to-operate strategy for the controlled and durable delivery of a vaccine against cancer. The design of the delivery system consisted of a self-healing nanocomposite hydrogel (NC) designed for the remote control of the tumor vaccine, which consisted of PLGA nanoparticles containing OVA as model antigen and imiquimod (R837), a member of the imidazoquinolinone family of synthetic immunostimulatory compounds that can be a dual agonist of TLR7/TLR8 [106], as adjuvant. The nanovaccine was named ORP-NP, which was afterward encapsulated into the NC gel, prepared from a precursor solution containing oligo (ethylene glycol) methacrylate (OEGMA) as the polymeric monomer and inorganic Laponite as physical cross-linker via hydrogen bonding to produce an injectable self-healing gel (ORP NC gel). The ORP NC gel was prepared by in-situ free radical polymerization using potassium persulfate as initiator and tetramethyl ethylenediamine as accelerator. The DC-cross presentation and maturation were evaluated in BMDC stimulated with OVA, OVA-PGLA, R837-PGLA, or OVA-R837-PGLA. ORP effectively activated DC, evidenced by high levels of cross-presentation of the SIINFEKL peptide by DC and upregulation of CD80 and CD86, as well as IL-6 and IL-12p70 induction. The prophylactic immunization scheme consisted of a single s.c. immunization with free ORP NP or ORP NC gel, using C57BL/6 mice. Moreover, another group was immunized with three (once a week) s.c. administrations of free ORP NP, an ultrasonic treatment was applied every two days (from 0 to 14 days). Finally, mice were challenged at day 21 with B16-OVA cells. The ORP NC gel with eight rounds of ultrasound stimulation effectively induced tumor reduction and augmented survival, compared to all other compounds tested. This response was mediated by SIINFEKL-MHC-I tetramer^+^ CD8^+^ T cells (CTL). In addition, this treatment induced IFN-γ secretion and the generation of NK and CD3^+^ CD8^+^ CD107a cells to greater extent than all other groups. The therapeutic scheme was evaluated in C57BL/6 mice that first received a s.c. inoculation of B16-OVA cells. Afterward, ORP NP or ORP NC gels were s.c. administered in sites adjacent to the tumors, with the ultrasonic treatment applied every two days (from 10 to 24 days). Moreover, an intradermal administration of α-PD-1 was applied every four days, starting at day 11 (up to day 27) post-immunization. They found that the ORP NC gel did not induce toxic effects, while delaying tumor growth and augmenting survival. This immune response was mainly induced by CD8^+^ CTLs and NK1.1 cells. Interestingly, the CD8^+^ CTLs/Treg ratio was strongly increased in the treatment that included the ORP NC gel, multiple ultrasound rounds, and administration of α-PD-1. In addition, mice from this group that had survived were challenged with B16-OVA cells on day 120, showing inhibition of tumor growth and survival increase up to day 160, this response was induced by CD3^+^ CD8^+^ CD62L^−^ CD44^+^ effector memory T cells, in conjunction with IFN-γ production (Figure 13). This response could be a promissory therapy to prevent tumor recurrence.

## 6. Discussion and Perspectives

The analysis of the current reports on nanoclay-based vaccine prototypes (a total of 14 reports), reveals that such nanocarriers exert adjuvant activity, increasing relevant immune responses with an acceptable safety. Nanoclays enhance the humoral and cellular responses to a variety of antigens from distinct origins, relative to use of antigens alone; thus, nanoclays have a remarkable potential for vaccinology. Infectious diseases caused by bacteria and cancer immunotherapy constitute the main explored targets with this vaccination technology and only two studies focused on viruses.

Among the pathways to expand this area is the discovery of the detailed mechanisms behind the adjuvant activity of nanoclays. The lack of systematic studies makes it difficult to compare the adjuvanticity of nanoclays with that of conventional adjuvants or other nanosystems. Therefore, a niche of opportunity is in performing a deep characterization of the global effects of nanoclays on immune system cells to propose hypotheses on the adjuvanticity mechanisms for this type of nanomaterial. Some groups have started such characterization, for instance it was found that LDH favors the maturation of DC and enhances cross-presentation of epitope/MHC class I complexes on the cell surface [92]. Proteomics and transcriptomics should be applied to depict, in detail, the pathways that these nanomaterials modulate. The fundamental knowledge from such studies will be critical to ultimately optimize nanoclays-based vaccines. The use of natural clays would be perhaps limited by variation in their composition. Therefore, a better characterization of their safety and the relationship with composition should be determined.

In terms of safety, only two studies determined the accumulation of clays in the administration site and only one case explored the carrier accumulation in key organs (lungs, liver, and kidney). In these works, nanoclays were deemed safe [57,85].

The evaluation of nanoclay-based vaccines has been primarily based on immunogenicity assessment and only in six reports it was assessed by performing infectious challenges. Importantly, among these six reports, only in one was non-significant protection found, reinforcing the potential of nanoclays to render highly effective vaccines [84], see Table 1. The achievement of long-term immune responses is critical for vaccine validation, a parameter that has been evaluated in three cases with promising findings at 3–4 months post-final immunization [56,86,89]. Although the use of nanoclays could ideally lead to self-adjuvanted vaccines, decorating the nanoclay conjugates with accessory adjuvant molecules is a possible approach to further enhance/optimize vaccine efficacy. CpG and other molecules targeting innate immunity receptors, which are critical to induce adaptive immunity, offer attractive adjuvant properties that could be systematically included in the vaccine design. In fact, multifunctional nanoclay-based conjugates have been proposed for cancer, by including in the nanocomplex, not only an immunostimulatory entity (CpG), but also a chemotherapeutic compound and a compound mediating photothermal therapy. This supports the notion of developing innovative vaccine prototypes, e.g., those targeting specific tumor-associated antigens or multiple antigens from infectious organisms, while at the same time carrying immunostimulatory compounds to target toll like receptors to enhance the innate immune response and ultimately increase immunogenicity [95].

Furthermore, it is always worth mentioning that nanoparticle technology, in this review centered on the generation of nanovaccines based on nanoclays, is always challenging in terms of successfully modifying the nanoclays’ surface with antigens, adjuvants, or ligands, while maintaining the stability of the nanoparticles as single entities. Once the surface of the nanoclays is modified with molecules of interest, the stability will certainly be affected (positively or negatively). In this regard, in the case of peptides or proteins with hydrophobic amino acid residues; once they are adsorbed on the surface of the nanoclays, they will favor the agglomeration and eventual aggregation of the nanoclay particles, due to the hydrophobic effect that is always favored by thermodynamics. Repulsive forces must be kept then upon functionalization, and they must surpass the attractive hydrophobic forces, especially when working with electrostatic stabilization. Steric stabilization has a better chance of keeping nanoclays as single entities. To the best of our knowledge, the use of polyethylene glycol (the most common polymer to provide steric stabilization) to first modify (before pursuing the passive or active adsorption of antigens, adjuvants, or ligands) the nanoclays’ surface has not been evaluated, nor has the effect that it will have in immunological studies. These two aspects are expected to be studied in the following years.

All the reports reviewed here deal with the passive adsorption of native or recombinant proteins on the surface of nanoclays to generate nanovaccines, in which these antigens must be added in large amounts, since they have a double function; they act as antigens and stabilizers of the nanosystem. Recombinant proteins are not generally produced with the high concentrations required for passive adsorption on nanoclays. Moreover, subunit vaccines using nanoclays to improve immunogenicity has not been reported. In this regard, having the ability to control the concentration of small peptides (the same is true for expensive, small ligands or adjuvants) through active (covalent) adsorption is a pending task. Covalent modification will be in line with the use of polyethylene glycol to impart stability, such that the small peptides only function as antigens. The best situation to explore, in this regard, is to use a bifunctional polyethylene glycol, in which one end of the molecule can be used to react with the nanoclay surface, while the other end is used to covalently attach a small peptide, ligand, or adjuvant. Active adsorption is expected to be explored in the following years and this review provides insights to the chemistry behind active adsorption on nanoclays.

The cost of vaccines not only depends on production and distribution, with the latter being a critical factor to ensure massive immunization, especially in poor or developing countries. Inorganic particles are often highly stable when compared to organic entities; therefore, nanoclays stand as promising candidates. However, no systematic evaluation of the stability of nanoclay-based nanovaccines is identified in references. Therefore, stability studies are required to better estimate the potential of clays to render thermally stable vaccines.

The administration route of nanoclay-based vaccines is another aspect that deserves research expansion to contribute to immune response optimization. Most of the studies were focused on parenteral administration of the test vaccines (14 of the reports), while only one case was based on mucosal administration (oral, see Table 1). Most of the current vaccines applied in human health are parenterally administered, which results in effective systemic IgG responses. However, the protective efficacy of the induced immune response against mucosal pathogens should be evaluated at the compartments through which the pathogen enters and propagates. In this way, vaccines will not only prevent the severe disease forms, but also provide sterilizing immunity (infection avoidance). Overall, the mucosal immunization routes offer desirable attributes, such as simple administration, noninvasiveness, flexibility, and the ability to trigger the common mucosal immune responses in the airways and the urogenital and gastrointestinal compartments. Therefore, mucosal immunization is an attractive approach to cope with the low compliance associated with painful administration, since oral and nasal administrations are more patient-friendly, while offering the possibility to induce a more effective immune response, by triggering a robust IgA response at mucosal compartments, thus increasing the possibilities to achieve sterilizing immunity. In fact, such an IgA response may occur in the site of administration, but also in distant mucosal compartments, thanks to the lymphocyte homing phenomenon that allows localizing antigen-specific cells in distant mucosal compartments. In addition, a systemic response (IgG) is often induced upon mucosal immunization.

The evaluation of nanoclay-based vaccines through mucosal immunization schemes deserves special attention. Moreover, combined schemes, comprising parenteral priming and boosting by mucosal routes are pertinent approaches to induce a balanced and robust immune response. In fact, this strategy is under exploration at clinical trials for anti-COVID-19 vaccines, where individuals are boosted by the airways after priming by the intramuscular route [107]. Interestingly, combining different mucosal routes has also been proposed to achieve protection in specific compartments, such as the gut [108].

In conclusion, nanoclays are promising carriers to design effective nanovaccines. However, the technology is still in its infancy and the coming decade will be critical to expand the exploration of their safety, perform mechanistic studies, optimize immunogenicity by assessing the inclusion of accessory adjuvants, and initiate clinical trials.

## Figures and Tables

**Table 1:** Main immunization results of nanovaccine prototypes based on clays.

Antigen	Clay	IR	Achieved Immunogenicity	Reference
Ovalbumin (OVA)	LDH	i.d.	The candidate nanovaccine induced a higher humoral response than the only-DNA vaccine. In addition, it showed protective immunity against a challenge. Moreover, it induced effective CTL activation and a Th1 immune response.	[82]
OVA and CpG ODN 1826	LDH	s.c.	The candidate induced significant antibody response, and promoted a switch from Th2 to Th1 response. In addition, it retarded tumor growth in a challenge after immunization.	[83]
rLipL32 from *Leptospira interrogans*	HNT	-	The nanovaccine candidate induced a significantly higher IgG response in comparison with the negative control. No protective immunity was provided against a challenge.	[84]
Intimin β (IB) from *E. coli*	LDH, HEC	s.c.	The nanovaccine candidates induced anti-IB IgG levels comparable to those induced with adjuvant. Interestingly, these IgG levels were maintained up to day 120. In addition, higher humoral immunity was recorded for LDH-IB.	[56]
(IB) from *E. coli*	HEC	s.c.	The candidate induced the strongest IgG immune response; in addition, it promoted the strongest sIgA secretion. Interestingly, HEC-IB can induce a cellular immune response.	[46]
Tyrosinase-related protein 2 (Trp2) and indoleamine 2,3-dioxygenase siRNA (siIDO)	LDH	s.c.	The candidate induced better tumor growth inhibition in mice immunized with the complete nanovaccine Trp2+LDH+siIDO (TLI) than other groups tested, including positive control groups. In addition, TLI induced significantly higher CTL.	[85]
IB from *E. coli*	LDH, HEC	s.c.	Potent cellular and humoral immune responses were induced in groups immunized with LDH-IB or HEC-IB in comparison with positive control groups. The candidate vaccines induced memory T-cell responses.	[57]
IB, proprietary antigen 1 (Pag1) and proprietary antigen 2 (Pag2) from *E. coli*	LDH, HEC	s.c.	The induction of IgG was more efficient in groups receiving LDH or HEC associated with the three antigens, sIgA antigen-specific levels increased at day 108. Similarly, an efficient cell immune response was induced in immunized groups receiving LDH or HEC associated with the three antigens.	[86]
CpG	LDH	i.v.	The candidate induced DC activation, CTL, and Th2 cells in situ and significantly inhibited the growth of primary and distant tumors. In addition, it significantly increased proinflammatory cytokine levels.	[87]
OVA and CpG	LDH, LDH NS	s.c.	The candidate nanovaccines induced strong Th1 and CTL immune responses, promoting inhibition of tumor growth and survivability.	[88]
rLemA from *Leptospira interrogans*	HNT	i.m.	After a challenge, the study revealed the induction of significantly higher IgG antibody response than the control groups. In addition, protective immune responses were observed.	[89]
Foot-and-mouth disease virus (FMDV)	LDH	s.c.	The candidate induced higher production of IFN-γ and anti-FMDV IgG antibodies than the positive control group. In pigs, similar levels of neutralizing antibodies were observed in LDH+FMDV and the positive control group, but higher than the negative control group.	[90]
OVA	LDH	i.v. and s.c.	The production of IgG antibodies was size-dependent; interestingly, similar levels of IgG1 and IgG2a were induced by the nanovaccine. The candidate showed total protection, mainly by CTL. In addition, the i.v. administration revealed efficient tumor inhibition.	[91]
Formalin-killed whole cells (FKC) of *Streptococcus agalactiae*	HNT	p.o.	HNT loaded with FKC induced an augmented humoral immune response in comparison with the control group, in a time dependent manner.	[92]

Abbreviations|i.d.: intradermal, s.c.: subcutaneous, i.v.: intravenous, i.m.: intramuscular, p.o.: per os (oral).
